# Microfluidic platform enables tailored translocation and reaction cascades in nanoliter droplet networks

**DOI:** 10.1038/s42003-020-01489-w

**Published:** 2020-12-14

**Authors:** Simon Bachler, Dominik Haidas, Marion Ort, Todd A. Duncombe, Petra S. Dittrich

**Affiliations:** Department of Biosystems Science and Engineering, ETH Zurich, 4058 Basel, Switzerland

**Keywords:** Nanofabrication and nanopatterning, Microfluidics

## Abstract

In the field of bottom-up synthetic biology, lipid membranes are the scaffold to create minimal cells and mimic reactions and processes at or across the membrane. In this context, we employ here a versatile microfluidic platform that enables precise positioning of nanoliter droplets with user-specified lipid compositions and in a defined pattern. Adjacent droplets make contact and form a droplet interface bilayer to simulate cellular membranes. Translocation of molecules across membranes are tailored by the addition of alpha-hemolysin to selected droplets. Moreover, we developed a protocol to analyze the translocation of non-fluorescent molecules between droplets with mass spectrometry. Our method is capable of automated formation of one- and two-dimensional droplet networks, which we demonstrated by connecting droplets containing different compound and enzyme solutions to perform translocation experiments and a multistep enzymatic cascade reaction across the droplet network. Our platform opens doors for creating complex artificial systems for bottom-up synthetic biology.

## Abstract (web summary)

Simon Bachler et al. present a new microfluidic platform to control the precise position and patterns of nanoliter droplets with various lipid materials. They show their platform enables monitoring of droplets and subsequent label-free mass spectrometry, which represents an important advance for the synthetic biology community.

## Introduction

Liposomes formed by synthetic lipids are widely used to mimic the compartmentalization of cells and create artificial cells. Like organelles in living cells, liposomes can serve as individual chemical reactors, in which biological processes can be performed. Essential compounds are retained in large concentrations, while others diffuse or are transported across the membrane^1–5^. The formation of lipid borders played a crucial role in the origin of life^6,7^ and therefore, liposomes are versatile models of early forms of life, so-called protocells.

One of the key challenges in the creation of artificial cells is the formation of a cell-like architecture, where different biochemical compounds are retained in smaller organelles within the cell and each organelle has a specific biological function. Many pathways require multiple steps in different organelles, translocation of intermediate products between organelles, and signaling cascades to enable the cell to sense the environment or relay information between different cellular regions. In this regard, major achievements were presented recently, reporting the realization of multistep reactions^4,5^, genetic circuits^8–10^, controlled division^11^ or communication pathways across membranes^12–14^ in artificial cell models.

Several experimental methods were introduced to form and connect multiple compartments. A particularly versatile approach uses water-in-oil emulsions, where a phospholipid monolayer is assembled at the interface between water and oil. Upon contact of two lipid-stabilized droplets, a so-called droplet interface bilayer (DIB) is formed^15–20^, allowing for the exchange of compounds from one droplet to the adjacent droplet by diffusion across the lipid bilayer or by translocation through membrane pores, e.g. alpha-hemolysin (α-HL)^21–25^. Two- and three-dimensional droplet networks and the encapsulation of droplets in droplets were reported to mimic cells and tissue^14,26,27^. These methods were greatly improved by the use of microfluidic methods instead of manual preparation and pipetting procedures^28^. Droplet microfluidics enables the formation of monodisperse droplets with precise volumes in the order of cell volumes, i.e., a few nanoliters, as well as efficient encapsulation of compounds and smaller liposomes. Droplets can be created and guided to specific locations where they are immobilized^29–32^. These systems, however, still have several shortcomings, such as the difficulty of supplying further compounds to selected immobilized droplets and the fact that droplets are not accessible for further analytical inspection. Furthermore, most of these closed microfluidic systems are prepared with polydimethylsiloxane (PDMS). Molecules can diffuse into PDMS, resulting in swelling of PDMS when common solvents like hexadecane are used. In addition, loss of analytes or water leads to droplet shrinkage and other instabilities^4,16,29,33^.

In this study, we build 1D and 2D networks of biochemical reaction compartments by automated deposition of droplets in close proximity and subsequent fast formation of stable DIBs on an open substrate, i.e., the substrate is not covered by a PDMS-based microfluidic system. The advantage of our method lies in controlling the initial droplet content and exact position on this substrate. Furthermore, we guide the translocation of molecules across selected droplets by the addition of the membrane pore α-HL. We can therefore selectively route small hydrophilic molecules that are otherwise membrane impermeable via α-HL pores. Our platform allowed for the creation of a multistep reaction, in which essential enzymes and compounds are initially separated in different compartments. The α-HL pores are used to connect selected compartments with each other.

Moreover, the use of an open platform overcomes additional limitations of commonly used microfluidic methods for DIB formation and analysis. Typically, assays with fluorescently tagged molecules or fluorogenic assays are required to study diffusion or reactions. Here, we show the detection of translocated molecules across DIB membranes by matrix-assisted laser desorption/ionization mass spectrometry (MALDI-MS), opening the use of the platform to a broad range of applications in the field of artificial cells, bioreactors, and pharmacological studies.

## Results

### Generation of droplet interface bilayer networks

**Fig. 1:**
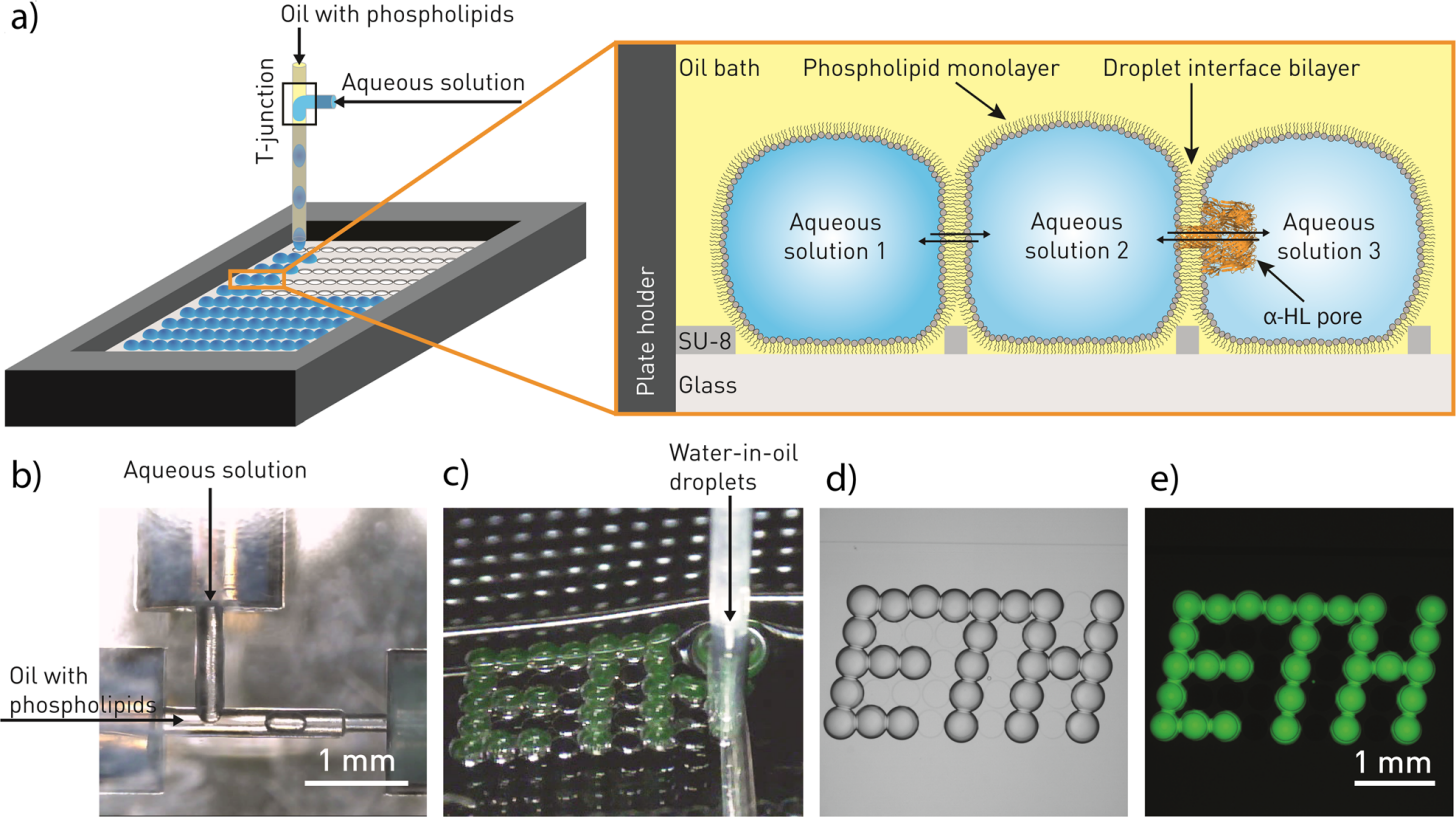
Concept of the microfluidic platform for artificial cell generation. **a** Schematics of droplet spotting and formation of the droplet interface bilayer (not to scale). Left: nanoliter droplets are formed in a microfluidic T-junction from an aqueous solution and oil with phospholipids. Thereafter, the droplets are transferred via a capillary and spotted on a glass plate with individual rows of cavities in close proximity. Phospholipids are present in the oil phase and self-assemble at the droplet interface while transitioning the capillary. Right: the spotted droplets are connected via a droplet interface bilayer. With our spotting platform, we can vary the content of the droplet next to each other and form droplet networks, here designated with aqueous solution 1-3. In addition, the pore forming toxin alpha-hemolysin (α-HL) can be selectively added to allow exchange of small water-soluble molecules between droplets. The α-HL structure was obtained from the Protein Data Bank (rcsb.org)^38^. **b** Photograph of the T-junction for droplet formation. **c** Spotting of 10 µM FITC-dextran in PBS droplets. **d** Brightfield image of the droplets spotted in **c**. **e** Fluorescent image of the droplets spotted in **c**.

Aqueous droplets of ~25 nanoliters were generated in hexadecane/squalane with dissolved lipids (by a microfluidic T-junction, Fig. 1a, b). The lipids self-assemble and form a lipid monolayer at the water oil interface during the transfer in the capillary^17,34^. The droplets were deposited with micrometer-precision on an SU-8 coated microscopy glass slide with cavities in close proximity. A predefined pattern can be spotted on the glass slide. Contact between adjacent aqueous droplets united the lipid monolayers and created a lipid bilayer membrane, which was visible through the microscope. The bilayer was fully formed after ~1 min at 37 °C. In this manner, droplets could be spotted in pairs, rows, and any other type of 2D pattern, as exemplified by the letters “ETH” in Fig. 1c–e (Supplementary Movie 1).

It should be noted that a mixture of hexadecane and squalane was used due to the long hydrocarbon length of these molecules, which improves the formation of a thin, oil-free DIB as reported also in former studies^29,35,36^. Moreover, the high stability of the DIB facilitated long-term experiments for at least 68 h.

### Pores in droplet interface bilayer networks

**Fig. 2:**
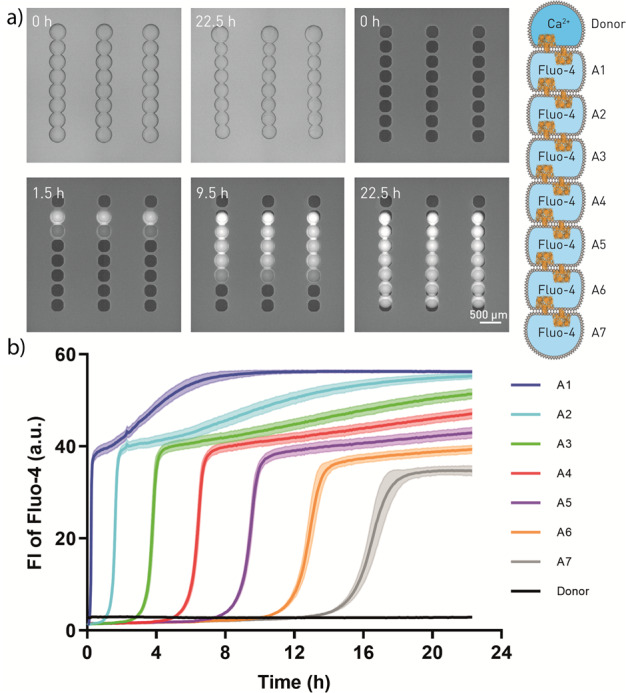
Translocation of Ca^2+^ across alpha-hemolysin (α-HL) pores in a droplet network. **a** Bright-field (top left and top middle) and fluorescent images of Ca^2+^ translocation across α-HL pores over multiple compartments. The top droplets are filled with Ca^2+^. The other seven acceptor droplets (A1-A7) in each row are filled with Fluo-4 pentapotassium salt, which is a membrane-impermeant calcium indicator. **b** Mean fluorescence intensity (FI) over time along the droplet network. Data were collected every ~5 min, *n* = 16 droplet networks. The error bands represent the standard deviation.

Next, we proved the formation of unilamellar DIBs by incorporation of α-HL. α-HL forms a transmembrane heptameric pore with a diameter of the channel between 1.4 and 2.4 nm in a lipid bilayer^37–41^. The pore formation facilitates the translocation of small molecules and ions such as Ca^2+^. It is general accepted that DIB membranes that host transmembrane toxins or proteins are virtually “oil-free” membrane structures similar to vesicles^37,42^. No translocation would be observed for multilamellar membranes since the toxin pore can only span a single lipid bilayer of physiologically relevant size. In a unilamellar lipid bilayer, on the other hand, the pore will create a passage for small molecules, which can then diffuse from one droplet into another along their concentration gradient^43^. Here, we show the reproducible formation of pores by using a row of eight droplets, each of them containing α-HL. Seven (acceptor) droplets were deposited, which contained Fluo-4 that forms a fluorescent complex with Ca^2+^ (Fig. 2). Last, a donor droplet with Ca^2+^ was placed at one end of the row. Upon diffusion of calcium through the pore, we can observe the increase of fluorescence intensity in the first droplet within 12–15 min and delayed in the following droplets (Fig. 2 and Supplementary Movie 2). In all droplets we found a characteristic two-phase time course. First, a rapid increase of fluorescence intensity where calcium ion influx results in the formation of the fluorescent complex was observed. Once the Ca^2+^ ions reach the next DIB and translocate into the next droplet, this efflux decreases the net influx and the fluorescence increase is slowed down. However, the same plateau height for all acceptor droplets was not reached after 22.5 h, which is both a consequence of the slow diffusion and the influence of osmolarity in the adjacent compartments. Although we carefully adjusted osmolarity in all droplets, the donor droplet volume increased over time, while acceptor droplets are visibly smaller. We believe this is due to the binding of calcium to Fluo-4 or EDTA in the acceptor droplets, which led to small changes (reduction) of the osmolarity, thereby promoting water permeation towards the donor droplet. At the same time, we did not observe Fluo-4 (731.60 Da) translocation towards the donor droplet for 100 U/mL α-HL. Calcium translocation does not occur without α-HL (SI Fig. S1) and droplet volumes were constant in this negative control. Higher α-HL concentrations of 500 U/mL (added in every droplet) resulted in droplet coalescence when also Fluo-4, EDTA, and Ca^2+^ were present. The high α-HL concentration and the osmolarity changes destabilized the DIB.

**Fig. 3:**
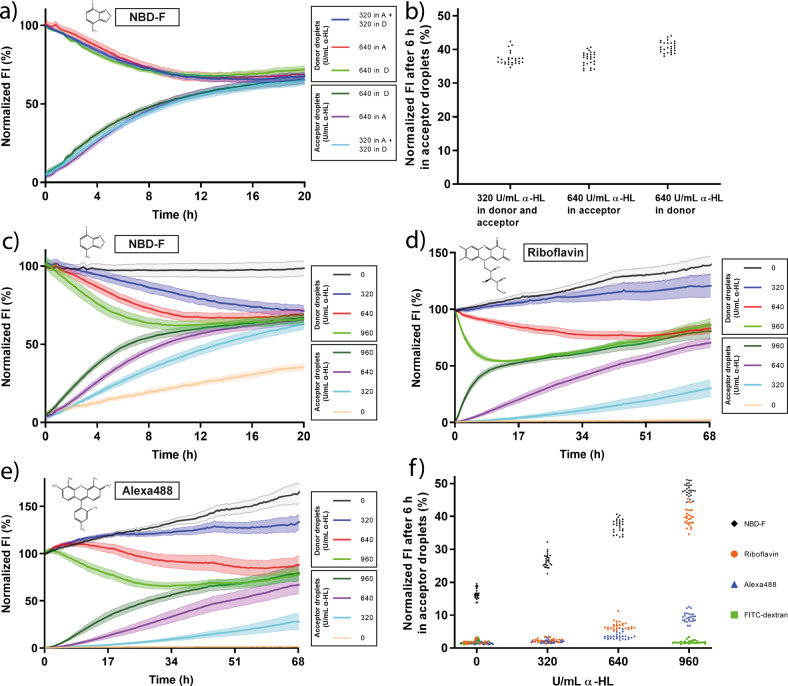
Translocation of NBD-F, riboflavin, and Alexa488 across alpha-hemolysin (α-HL) pores. **a** Mean fluorescence intensity (FI) over time of 300 µM NBD-F translocation across α-HL pores from donor (D) to acceptor (A) droplets. Comparison of 640 U/mL α-HL in acceptor, 640 U/mL α-HL in donor, or 320 U/mL α-HL in acceptor and donor droplets. Data were collected every ~15 min, *n* = 27 droplet networks for every curve. **b** Mean FI of NBD-F after 6 h in the acceptor droplets. **c**–**e** Mean FI over time for translocation of the fluorophores NBD-F (300 µM, **c**), riboflavin (60 µM, **d**), and Alexa488 (60 µM, **e**) at different concentrations of α-HL. Here, the numbers in the labels refer to the α-HL in the acceptor droplets, no α-HL was added to the donor droplets. *n* = 27 droplet networks for every curve. **f** Mean FI of 300 µM NBD-F, 60 µM riboflavin, 60 µM Alexa488, and 60 µM FITC-dextran (0 and 960 U/ml α-HL) after 6 h in the acceptor droplets. The error bands represent the standard deviation in all figures. The continuous increase in the normalized FI in the experiments can be attributed to slow droplet shrinkage over time. We only normalized to the starting fluorescence of the individual donor droplets and did not account for droplet shrinkage in the data normalization process.

After the successful translocation of calcium, we investigated the influence of pore direction, i.e., we supplied α-HL either in the donor or in the acceptor droplet. For this experiment, we spotted two droplets. The donor droplet contained the small hydrophobic fluorescent molecule NBD-F (mass of 183.10 Da). Either the donor or the acceptor additionally contained α-HL (640 U/mL), or both droplets contained α-HL at a concentration of 320 U/mL. Fluorescence intensity was monitored in both droplets over time, as shown in Fig. 3a, b. No difference in translocation velocity was observed for the same amount of α-HL in the bilayer membrane. Increasing the α-HL concentration accelerated the translocation as more pores were formed (Fig. 3c). An equilibrium was reached with 960 U/mL α-HL for NBD-F after ~12 h. In all experiments with NBD-F, DMSO was added to the aqueous droplet to avoid leakage of NBD-F into the oil^44^. NBD-F also permeates slowly across the membrane when no α-HL was present (Fig. S2).

Next, we tested the translocation of riboflavin with a mass of 376.37 Da (Fig. 3d) and Alexa488 with a mass of 531.44 Da (Fig. 3e) to demonstrate the dependence of the translocation process on molecule size and α-HL concentrations. An equilibrium was reached with 960 U/mL α-HL for riboflavin after ~18 h and for Alexa488 after ~48 h, as defined by the donor and acceptor droplets having equal fluorescence intensities.

The continuous increase in fluorescence intensity in these experiments can be attributed to slow, but continuous droplet shrinkage, and therefore an increase in local fluorophore concentration over time. We observed very slow droplet shrinkage over time in all experiments, which was only clearly visible for experiments over 2 days. For example, for the Alexa488-filled droplets in Fig. 3e, the diameter decreased down to ~70% of the original diameter within 68 h (SI Fig. S3A).

For a large molecule like FITC-dextran (~70 kDa), no translocation across α-HL pores (960 U/mL) was observed within 68 h (Fig. S4). Since we did not observe translocation of FITC-dextran through the pore with the diameter of 1.4–2.4 nm, we would also not expect translocation of other large molecules like proteins across α-HL pores in our system. Figure 3F compares the fluorescence intensity of NBD-F, riboflavin, Alexa488, and FITC-dextran after 6 h in the acceptor droplets. In addition, we calculated the translocation rates (SI Table S1), which confirmed the fastest rate for NBD-F at the highest α-HL concentration (180.9 × 10^−3^/h), compared to the rates for riboflavin (137.0 × 10^−3^/h) and Alexa488 (38.3 × 10^−3^/h).

### Label-free molecule detection in droplet interface bilayer networks

**Fig. 4:**
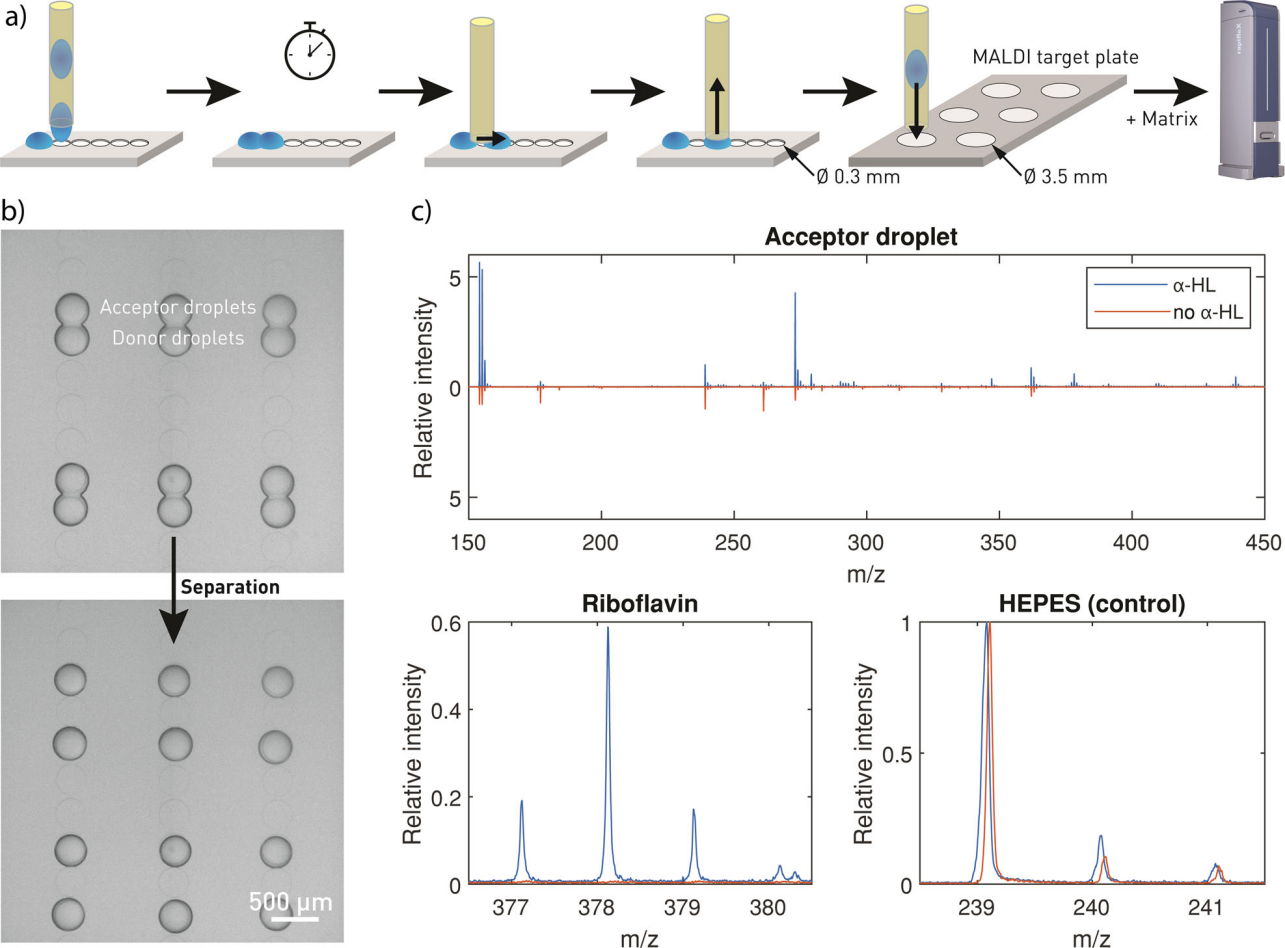
Label-free detection of translocation across alpha-hemolysin (α-HL) pores with mass spectrometry. **a** Scheme of droplet separation and extraction for MALDI-MS analysis (not to scale). From left to right: droplet spotting; incubation step for allowing translocation; droplet separation; droplet aspiration; placing the droplet on a stainless-steel MALDI target plate; and MALDI-MS analysis after matrix (DHB) deposition. **b** Droplets connected by a droplet interface bilayer (top) and droplet pairs after separation (bottom). **c** MALDI-MS analysis of accepter droplets, where the donor droplet contained riboflavin (377.1 *m*/*z*) and the acceptor droplet contained α-HL (blue) or not (orange, here flipped for better visualization). Data are shown for *n* = 1 droplet network. The isotopic pattern for riboflavin^53^ and HEPES, again with (blue) and without (orange) addition of α-HL. Each spectrum is normalized to the intensity of the internal control HEPES (239.1 *m*/*z*).

Monitoring of processes at and across DIBs is frequently conducted by means of fluorescence methods, using fluorescently labeled compounds or by the implementation of fluorogenic assays. With the aim of overcoming this limitation, we developed a protocol to interface our platform with MALDI-MS for label-free detection of translocation across the DIB (Fig. 4a). Droplets were spotted, incubated to allow translocation, separated, and extracted for final measurements by MALDI-MS. Central in this process is the step to separate droplets, which we successfully realized by placing the capillary between the two connected droplets. This initiated the movement of the droplet to the next hydrophilic cavity (Fig. 4b), from where it could be extracted by the capillary and transferred to the MALDI plate. After the addition of the matrix, we could analyze the content of the dried droplet. Riboflavin and HEPES were detected by MALDI-MS in the acceptor droplet when α-HL was present (Fig. 4c). In the acceptor droplet without α-HL, only HEPES was detected. Therefore, the riboflavin translocation across the α-HL pore was confirmed with MALDI-MS in addition to fluorescence microscopy (Fig. 3d). This MALDI-MS readout approach opens the possibility to probe the translocation of non-fluorescent compounds. In the supplementary information, we included the results for the label-free translocation of l-arginine (SI Fig. S5) and l-histidine (SI Fig. S6) across α-HL pores.

### Cascade reaction in droplet networks

**Fig. 5:**
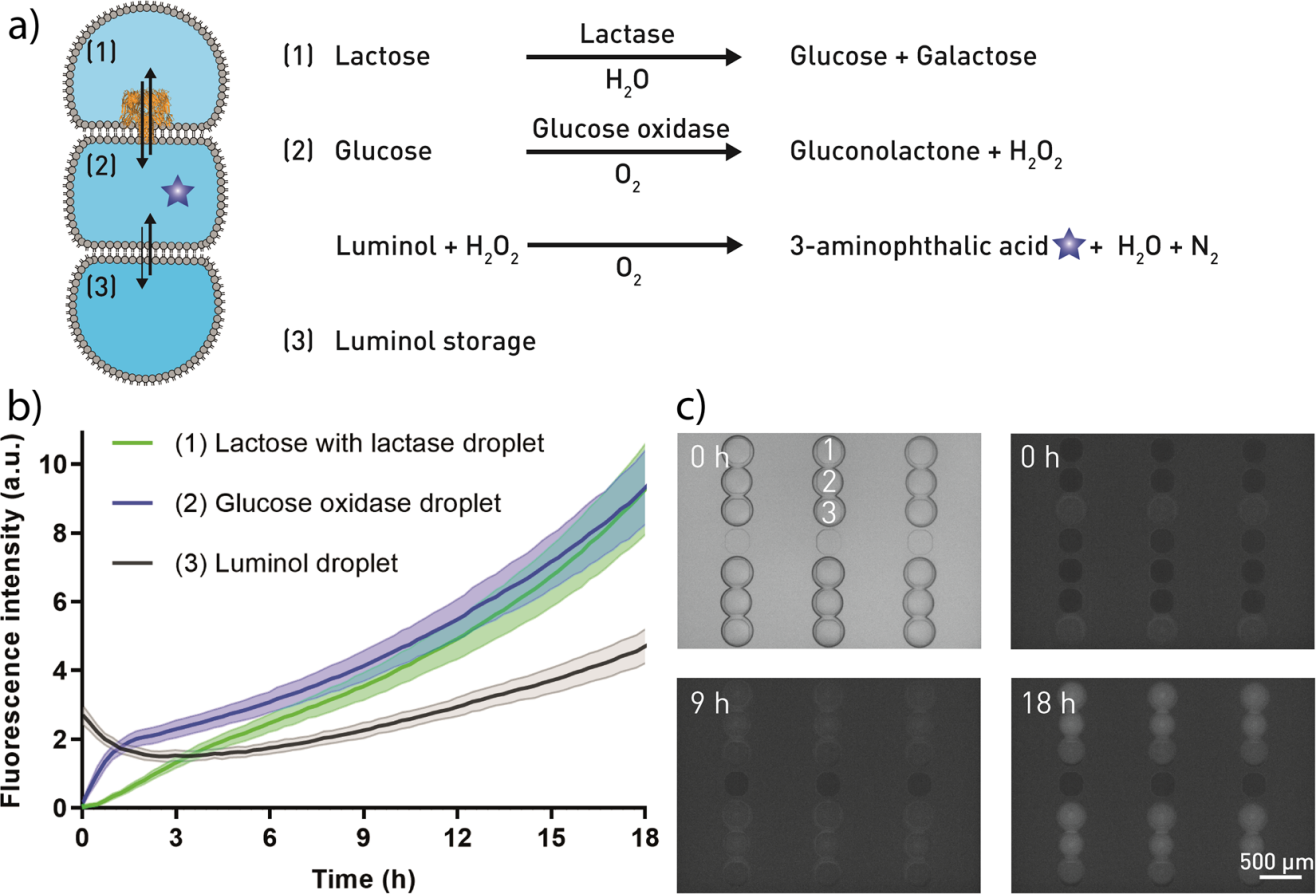
Compartmentalized enzymatic cascade reaction. **a** Sketch of the three-step reaction (not to scale). Lactase hydrolyses lactose to glucose and galactose (1). Small molecules i.e. glucose translocate across alpha-hemolysin (α-HL) pores to the next droplet (2), where glucose oxidase catalyzes the oxidation of glucose to hydrogen peroxide and gluconolactone. Luminol permeates from an adjacent droplet (3) into the other ones. Upon contact with hydrogen peroxide, luminol forms the highly fluorescent molecule 3-aminophthalic acid. **b** Mean fluorescence intensity of the reaction in **a**, **c** in dependency of the time. Data were collected every ~15 min, *n* = 30 droplet networks. The lactose/lactase droplet (1) was added to the glucose oxidase droplet (2) ~80 min before the luminol droplet (3). The imaging starting point was directly after spotting of the luminol droplet. The error bands represent the standard deviation. **c** Bright-field (top left) and fluorescent images of the reaction in **a**/**b**. Lactose and lactase with α-HL in the top droplet (1). Glucose oxidase in the middle droplet (2). Luminol in the bottom droplet (3).

Finally, we demonstrate with our droplet networks a compartmentalized enzymatic cascade reaction, where each droplet of the network contains different compounds and enzymes (Fig. 5a). The final conversion of luminol to the fluorescent product 3-aminophthalic acid occurred in the central droplet. Luminol was stored in an adjacent droplet, from where it permeated across the DIB. The reaction requires hydrogen peroxide, which was formed during the conversion of glucose to gluconolactone by glucose oxidase. Glucose translocated via α-HL from the adjacent droplet, where it was formed from lactose in the presence of β-galactosidase (lactase).

Once all droplets were spotted and the DIBs were formed, the reactions began and the fluorescent product became detectable. The product immediately translocates via the α-HL pore into the adjacent droplet, therefore we observed a rapid increase of fluorescence intensity in these two droplets (Fig. 5b, c and Supplementary Movie 3). The fluorescence intensity increased slowly in the original droplet with luminol due to the passive permeation of the product. Without α-HL or lactase the fluorescence increase was not observed (SI Figs. S7 and S8) confirming that all steps of the reaction cascade are required to obtain the fluorescent product.

## Discussion

We present a platform of tailored 1D or 2D networks of nanoliter droplet microreactors interconnected by DIBs as an analytical model for studying multi-compartmental artificial cells. Arrayed droplets function as discretized reactors containing selected reagents. Deliberate selection of membrane composition and functional transport units at the droplet-droplet interface enables the control of chemical communication throughout the droplet network. Monitored by real-time fluorescence microscopy and endpoint MALDI-MS, our interconnected droplet networks offers a powerful tool for researchers studying interactions of molecules with lipid membranes such as permeation and pore formation. DIBs are stable over days, and the droplets shrink very slowly within days. This is a strong improvement for long-term studies compared to DIBs created in PDMS devices, where droplets must be stabilized by coating of the PDMS walls or the addition of on-chip water reservoirs to prevent diffusion of the aqueous droplet content into the PDMS^4,45,46^.

The current size of the droplets can be reduced to sizes of mammalian cells (15–20 µm) by creating droplets with other methods than the used T-junction. Also the spotting plate is easily scalable to provide smaller droplet deposition sites (theoretically down the resolution of photolithography, i.e. ∼1–2 µm), or to increase the number of deposited droplet pairs and networks up to several hundred thousand. In principle, we can spot arbitrary arrays of networks; however, the number of droplets with different content is limited in the current system as we have to change the supplied fluids in the syringes manually.

In our study, we insert the membrane pore α-HL to enable the transfer of small water-soluble molecules between two adjacent droplets. By adding α-HL to specific droplets we can selectively route small hydrophilic molecules across lipid membranes while translocation is restricted without α-HL.

Our complex droplet networks are directly generated with precision spotting on an open substrate. In addition to the ease of addressing individual droplets with spotting, the open array allows us to interface the platform with other analytical techniques such as mass spectrometry. This greatly increases the possibilities for detection of molecules in DIBs compared to fluorescence microscopy and closed microfluidic systems. Further, we are able to stop translocation and disrupt the DIBs by separating droplets, a feature that is not possible with more common techniques for studying DIBs. This could be exploited to stop supplies to the adjacent droplet e.g. for an enzymatic reaction.

We demonstrated the versatility of our platform by the creation of a cascade reaction, in which reactants and enzymes are initially separated in different compartments. We used α-HL pores selectively to tailor the multistep reaction for allowing passage of glucose and no pores were inserted in the other membrane to create a barrier for molecules, which are impermeable or very slowly permeating.

The presented droplet networks simulate chemical communication across a long network of researcher-designed microreactors and membranes. This is well suited for conducting multistep reactions, in which each droplet contains different essential compounds or enzymes. Moreover, we demonstrate that the content analysis of droplets can be achieved by mass spectrometry, allowing for the multiplexed analysis of unknown and/or unlabeled compounds. We believe this opens the door for experiments with DIBs, where fluorescence labeling is not possible or not preferred. In the future, the platform can be employed for other membrane studies, e.g., it facilitates transporter or permeation studies as required for drug discovery and drug testing with asymmetric membranes. Our platform could mimic a simple cell/organelle model such as a mitochondria membrane in a cell, or artificial cell clusters or tissue.

## Methods

### Fabrication of the microarray plates with cavities

A 4-inch borofloat glass wafer was cleaned in an oxygen plasma for 5 min (plasma asher system 200, TePla, Germany). Immediately afterwards, SU-8 3025 negative photoresist (MicroChem, Westborough, MA, USA) was spin-coated to obtain an ~35 μm high layer of photoresist (dynamic spread at 500 rpm for 10 s with 100 rpm/s and spin at 2500 rpm for 30 s with 300 rpm/s). Subsequently, after ~3 min at room temperature, the wafer was soft baked at 65 °C for 2 min and 95 °C for 12 min. Following this, we used a mask aligner (MA8BA8-Gen3, SÜSS MicroTec, Garching, Germany) to expose the photoresist to a UV light source through a foil mask with images of the desired cavity arrays (i-line illumination with 270 mJ/cm^2^). For the post-exposure bake, we used a ramp from room temperature to 95 °C over 60 min, held at 95 °C for 5 min, and cooled down again to room temperature over 60 min. To form the cavities, we developed the wafer with mr-Dev 600 (micro resist technology GmbH, Berlin, Germany) for 6 min, followed by rinsing with 2-propanol (Technic France, Saint-Denis, France) for another 10 s, and spin-drying. We conducted a hard bake with a ramp over 4 h to 180 °C, incubated at 180 °C for 2 h, and cool down to room temperature over 4 h. Using ramps was important for the post-exposure bake and hard bake to avoid SU-8 detachment and cracks in the resist layer. Finally, the wafer was diced in two 75 mm × 25 mm microarray plates with cavities (the “droplet deposition sites”). The final microarray plates host 2520 spots (plates for networks) or 1512 spots (plates for droplet pairs) for droplet deposition. To achieve the formation of the DIBs between two droplets, adjacent cavities are positioned at a distance of 310–330 µm.

### Droplet spotting platform and operation

The microarray plate was placed on a removable temperature-controlled holder with a transparent bottom (quartz microscope slide of 0.5 mm thickness, Electron Microscopy Sciences, Hatfield, PA, USA), which was mounted on a motorized XY stage (HLD117, Prior) of an inverted fluorescence microscope (Olympus IX73). The holder wall prevents oil leakage, which allowed for carrying out the experiments in an oil bath to reduce droplet evaporation^47^.

For droplet formation, 1 mL syringes (309628, BD, Heidelberg, Germany) were filled with oil and aqueous solutions, mounted on syringe pumps (Nem-B101-03A, Cetoni, Korbußen, Germany), and connected to a self-made microfluidic T-junction device (material: polycarbonate) using polytetrafluoroethylene (PTFE)-tubing of 250 μm inner diameter (PKM SA, Lyss BE, Switzerland) and polyether ether ketone (PEEK)-connectors (F-120 10-32 PEEK Fitting and P-659 Female Luer to 10-32 Female; Ercatech AG, Bern BE, Switzerland). The T-junction was coated with 1H,1H,2H,2H-perfluorodecyltrichlorosilane (ABCR-Chemicals, Karlsruhe, Germany) carried by a nitrogen stream for ~20 min to prevent wetting of the channel walls by the aqueous phase and to ensure stable droplet generation. Droplets of several nanoliters were generated in the T-junction by injecting the aqueous phase (flow rate: 0.5 μL/min) into the immiscible oil phase (flow rate: 2 μL/min), and was monitored by a CCD camera (AD-3713TL, Dino-Lite).

The T-junction diameter is 300 µm that is reduced to 200 µm at the exit and further reduced to 150 µm in the HPFA + capillary (1933, IDEX Health & Science SA, Glattbrugg ZH, Switzerland), which is used after the T-junction to transport the droplets to the microarray plate. During droplet transit, the phospholipids in the oil aligned along the water/oil interface and formed a monolayer^1,2^.

The end of the capillary was mounted on a motorized Z stage (M-403.2PD, Physik Instrumente, Karlsruhe, Germany) and positioned just above the microarray plate. A custom-made optical droplet detector positioned shortly before the end of the capillary^48^ was used to synchronize droplet, capillary and microarray movement to selectively deposit single droplets per position. The software YouScope (R2018, v2.1.0) controlled all components of the microscope and the capillary for this automated workflow, where we spotted at frequencies of 0.2–0.4 Hz^49^.

Before spotting of the droplets, the plate holder was filled with ~4 mL of a 1:1 (v/v) hexadecane/squalane mixture (without phospholipids, *T* = 37 °C) and 50 µL water in all four edges to reduce droplet shrinkage. For the experiments over 68 h, twice 100 µL water was added to the plate holder in similar manner after the first and second day. The generated droplets were detected in the capillary holder with an optical droplet detection system consisting of a red light-emitting diode (LED Fiber Optics 660 nm Superbrite Red, IF-E97, Industrial Fiber Optics, Tempe, AZ, USA), a polymeric plastic fiber-optic cable (198412, Conrad Electronic SE, Hirschau, Germany), and a phototransistor (IF-D92, Industrial Fiber Optics). The light from the LED was coupled into the optical fiber and the photodetector attached to the other side of the optical fiber was used to detect changes in the transmittance between the aqueous and the oil phase inside the capillary. The signals were recorded and processed by a real-time data acquisition system (AdWin Gold II, Jäger Computergesteuerte Messtechnik GmbH, Lorsch, Germany). The system allowed for smoothing of the raw data and threshold analysis to differentiate droplets from oil plugs. Signals were collected by a custom-made Matlab-based script (MathWorks, Natick, MA, USA) integrated in the control software YouScope. When a droplet was detected, the Z stage raised the capillary 2 mm up, the microscope stage moved the center of the capillary over to the next spot, after which the Z stage lowered the capillary 2 mm downwards. Exceptionally, the up and down movement of the capillary for spotting the letters “ETH” was 0.3 mm. The movement was performed while an oil plug was exiting the capillary, and the final spotting height was 300 μm above the plate. Typical spotting frequency was 0.2–0.4 Hz. Droplets were spotted precisely on individual cavities with 300 µm diameter and a depth of ~35 µm, which we monitored with a second CCD camera (AD-3713TL, Dino-Lite). The distance between two neighboring cavities was 310–330 µm. When two droplets were spotted directly next to each other, the phospholipid monolayers at the droplet surfaces touched each other, and a droplet interface bilayer formed.

### Lipid solution preparation

We used the lipid-out approach by adding phospholipids to the interface from the oil phase. 1,2-diphytanoyl-sn-glycero-3-phosphocholine (DPhPC, Avanti Polar Lipids, Alabaster, AL, USA) was purchased as a solution in chloroform. We transferred the appropriate amount of lipid solution into a pear-shaped flask and removed the solvent with a rotary evaporator (Büchi Labortechnik AG, Flawil, Switzerland). The remaining lipid film was dissolved in hexadecane (Reagent plus grade, Sigma-Aldrich) and squalane (Sigma-Aldrich) (1:1 (v/v)), if not stated otherwise, while being agitated by an ultrasonication bath at 50 °C for ~30 min. The final phospholipid concentration in oil was 0.5–5 mg/mL. Before use, the oil solution with phospholipids was filtrated (0.45 μm pore size, RC 4 Male Luer Slip Minisart filters, Huberlab, Switzerland).

### Assays

All aqueous solutions were prepared in ultrahigh-purity water (Barnstead GenPure Pro, Thermo Scientific, Niederelbert, Germany), if not stated otherwise. The osmolarity of all solutions for donor and acceptor droplets were matched and verified with an Osmomat 3000 (gonotec, Berlin, Germany).

Calcium translocation experiments were carried out with 1 M calcium chloride dihydrate (CaCl_2_, Sigma-Aldrich), 100 U/mL α-HL from *Staphylococcus aureus* (Sigma-Aldrich), 20 µM ethylenediaminetetraacetic acid (EDTA, Titriplex II, Merck, Darmstadt, Germany), and 10 mM 4-(2-hydroxyethyl)-1-piperazineethanesulfonic acid (HEPES, pH 7.4, gibco, Paisley, UK) in the donor droplet. We used 1.5 M potassium chloride (KCl, Reagent plus grade, Sigma-Aldrich), 100 U/mL α-HL, 10 µM Fluo-4 pentapotassium salt (Life Technologies, Eugene, OR, USA), 200 µM EDTA, and 10 mM HEPES in the acceptor droplets. For this experiment, we used 100% hexadecane without squalane to dissolve the lipids, but hexadecane/squalane in a 1:1 mixture as oil bath identical to all other experiments. No α-HL was used for the negative control.

We spotted the letters “ETH” with droplets consisting of 10 µM fluorescein isothiocyanate-dextran (FITC-dextran, 70 kDa, Sigma-Aldrich) in phosphate buffered saline (PBS, pH 7.4, gibco, Paisley, UK).

For the 4-fluoro-7-nitrobenzofurazan (NBD-F) translocation experiments, we used acceptor droplets with 0, 320, 640, or 960 U/mL α-HL. The donor droplets consisted of 0.3 mM NBD-F (Sigma-Aldrich, Switzerland). To test if there was a difference whether the α-HL pore was inserted from the donor or acceptor droplet side, we used 0.3 mM NBD-F in combination with 640 U/mL α-HL in the donor, 640 U/mL α-HL in the acceptor, or 320 U/mL α-HL in both acceptor and donor droplet. All droplets in the experiments with NBD-F were filled with 10 mM HEPES and 5% dimethyl sulfoxide (DMSO, Sigma-Aldrich).

The riboflavin, Alexa488, and FITC-dextran translocation experiments were conducted with 10 mM HEPES with 0, 320, 640, or 960 U/mL α-HL as acceptor droplets. For the donor droplets, we used 10 mM HEPES in combination with 60 µM riboflavin (Sigma-Aldrich), Alexa Fluor 488 carboxylic acid (Alexa488, Life Technologies), or FITC-dextran.

For the label-free measurements, the following solutions were dissolved in LC-MS grade water (Fisher Scientific, Loughborough, UK). We used 20 mM HEPES in all acceptor droplets with 0 or 400 U/mL α-HL. The donor droplets consisted of 10 mM HEPES and 10 mM L-arginine (BioUltra grade, Sigma-Aldrich) or 10 mM HEPES and 10 mM L-histidine (Reagent plus grade, Sigma-Aldrich), or 20 mM HEPES and 200 µM riboflavin.

All solutions for the enzyme cascade assays were matched to ~300 mOsmol/kg with potassium chloride. We incubated 10 U/mL β-galactosidase (lactase) from *Escherichia coli* (Sigma-Aldrich) together with 50 mM lactose monohydrate (Hänseler, Herisau, Switzerland), and 60 mOsmol/kg PBS overnight. The spotted lactase droplets had a final concentration of 5 U/mL lactase, 25 mM lactose monohydrate (converted), 10 mM tris(hydroxymethyl)aminomethane (tris, pH 9, VWR Life Science), 400 U/mL α-HL, and 30 mOsmol/kg PBS. The glucose oxidase (GOx) droplets consisted of 5 U/mL GOx from *Aspergillus niger* (Sigma-Aldrich), 10 mM tris, and 30 mOsmol/kg PBS. The luminol droplets consisted of 4 mM luminol (TCI, Tokyo, Japan), 10 mM tris, and 30 mOsmol/kg PBS. For the negative controls no α-HL or no lactase were used.

### Imaging

After droplet spotting, brightfield and fluorescence pictures were recorded using a light source (TH4-200, Olympus and Lumen 300, Prior) and a CMOS camera (Zyla 4.2, Andor) connected to the Olympus IX73 microscope. To fluorescently track luminol, a UV-excitation filter set (exciter BP H 365/12, dichroic BS DCLP 395, and emitter 397 LP H; Delta optical thin film, Hørsholm, Denmark) was used. For FITC-Dextran, Fluo-4, NBD-F, riboflavin, and Alexa488 a blue excitation filter set (exciter HQ470/40x, dichroic 500dcxr BS, and emitter E515lpv2; Chroma Technology Corp, Bellows Falls, VT, USA) was used. A black anodized lid was placed on top of the plate holder for the fluorescence pictures to minimize environmental influences and heat exchange.

### Data evaluation and statistical information

The recorded fluorescence signals were evaluated using Fiji^50^ and a custom Matlab (R2017b, MathWorks) script, and Prism (8.3.0, GraphPad). For the normalized fluorescence intensity values, 100% was the starting fluorescence of the individual donor droplets in the first image. We only normalized to the starting fluorescence and did not account for droplet shrinkage (except SI Fig. S3B). The background fluorescence was subtracted for data evaluation. For the translocation experiments analysis with NBD-F, riboflavin, Alexa488, and FITC-dextran, we selected the first 27 droplet networks. Droplets that did not touch each other in a network on the first image were excluded. We used the Grubbs’ test (extreme studentized deviate method) to determine whether the most extreme value in the list was a significant outlier from the rest (*ɑ* = 0.05, two-tailed, Outlier calculator, QuickCalcs, GraphPad).

### Droplet separation and extraction

For spotting experiments with subsequent MALDI-MS analysis, the droplet pairs were separated and extracted using a custom-made YouScope script. The capillary was flushed with hexadecane/squalane (1:1) and positioned 1300 µm above the plate. The center of the capillary was placed between the two connecting droplets directly over the droplet interface bilayer. To separate two adjacent droplets, the capillary was moved in Z direction to 50 µm above the plate. When this position was reached, the capillary moved 150–170 µm in the desired Y direction to push one droplet into a neighboring cavity. Thereafter, the capillary was raised to the original level of 1300 µm above the plate. We introduced several waiting steps between the different moves to prevent the droplets from sliding back together.

After separating all desired droplet pairs, we flushed the capillary with fluorinated oil (HFE-7500, 3 M Novec, Hadfield, UK) and connected it to a 50 µL glass syringe (Hamilton, Switzerland). The center of the capillary was positioned directly over the center of the droplet and lowered to 150–250 µm above the plate to squeeze the droplet. The droplet was aspirated by slowly pulling the glass syringe. As soon as the droplet was aspirated, the capillary was raised above the oil level to minimize the hexadecane/squalane aspiration as much as possible. Afterwards, the capillary was moved over a ground steel BC MALDI target plate (Bruker Daltonik GmbH, Bremen, Germany) and the aspirated droplet was ejected on a spot. The MALDI target plate was heated to 120 °C for 5 min to induce water/solvent evaporation.

### MALDI-MS

We added 0.2 µL of 20 mg/mL 2,5-dihydroxybenzoic acid (DHB, Sigma-Aldrich) in 70% LC-MS grade water with 0.1% trifluoroacetic acid (Acros Organics) and 30% acetonitrile (VWR) to the individual spots on a ground steel MALDI target plate. Mass spectra were acquired from 140 *m/z* to 460 *m/z* using a Bruker rapifleX MALDI-TOF/TOF in positive reflector mode (Bruker Daltonik GmbH). Each mass spectrum consisted of 1000 shots performed in a 10-µm square region using a 10-kHz laser repetition frequency. Mass spectra were baseline subtracted and calibrated to DHB matrix ions with the mMass software tool^51^. For comparisons between donor and acceptor droplets, intensity values were normalized to the internal standard peak of HEPES (239.1 *m/z*). The detected and theoretical monoisotopic peaks are compared in Table S2 for analytes l-histidine, l-arginine, HEPES, and riboflavin. All detected analyte peaks had an *m/z* error of <0.025.

### alpha-Hemolysin structure

The α-HL structure was obtained from the Protein Data Bank (rcsb.org)^38,52^ and visualized with Jmol (an open-source Java viewer for chemical structures in 3D. jmol.org).

### Reporting summary

Further information on research design is available in the Nature Research Reporting Summary linked to this article.

## Supplementary information

Supplementary Information

Description of Additional Supplementary Files

Supplementary Movie 1

Supplementary Movie 2

Supplementary Movie 3

Reporting Summary

## Data Availability

The datasets generated and/or analyzed during the current study are available from the authors on reasonable request.
