# Supplementary material for: Microfluidic platform enables tailored translocation and reaction cascades in nanoliter droplet networks
Source: Commun Biol. 2020 Dec 14;3:769. doi: 10.1038/s42003-020-01489-w (PMC7736871; doi:10.1038/s42003-020-01489-w)
Supplement: Supplementary file 1 — Supplementary Information [file 42003_2020_1489_MOESM1_ESM.pdf]

# Supplementary information:

# Microfluidic platform enables tailored translocation and reaction cascades in nanoliter droplet networks

Simon Bachler, Dominik Haidas, Marion Ort, Todd A. Duncombe, Petra S. Dittrich*

Department of Biosystems Science and Engineering, ETH Zurich, 4058 Basel, Switzerland

*Corresponding author: petra.dittrich@bsse.ethz.ch

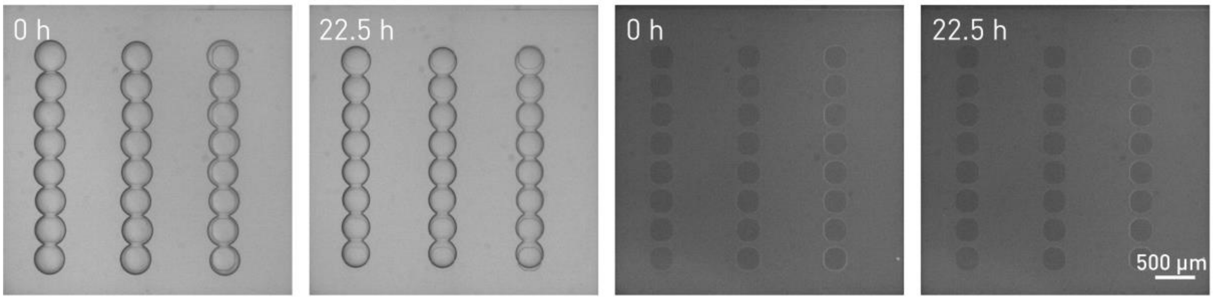


*Figure S1: Negative control for the $Ca^{2+}$ translocation and Fluo-4 experiment without α-HL. We did not detect $Ca^{2+}$ translocation across the droplet networks when no α-HL was present (N = 45 droplet networks).*

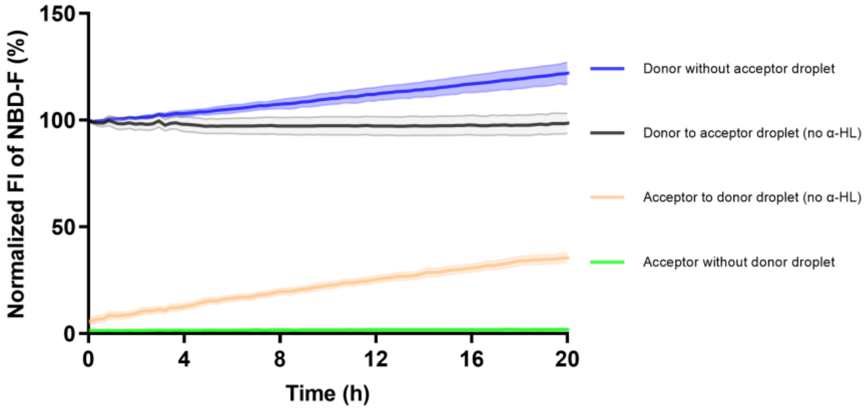


*Figure S2: Mean fluorescence intensity (FI) over time of NBD-F diffusion across the membrane (black and yellow curves) in comparison to acceptor without donor droplets (green curve) and donor without acceptor droplets (blue curve). Data were collected every ~15 min, N = 27 droplet networks for every curve. The increase in FI occurred (blue curve), because the droplets shrank over time to a small extent and the concentration of the local fluorophore increased. NBD-F slowly permeated across the membrane also when no α-HL was present. No NBD-F passage over oil with 5% DMSO was observed as can be seen by the flat FI curve for the acceptor not connected to donor droplets (green curve) in the same oil bath as by the experiment with several donor droplets in a distance of a few millimeters. The error bands represent the standard deviation.*

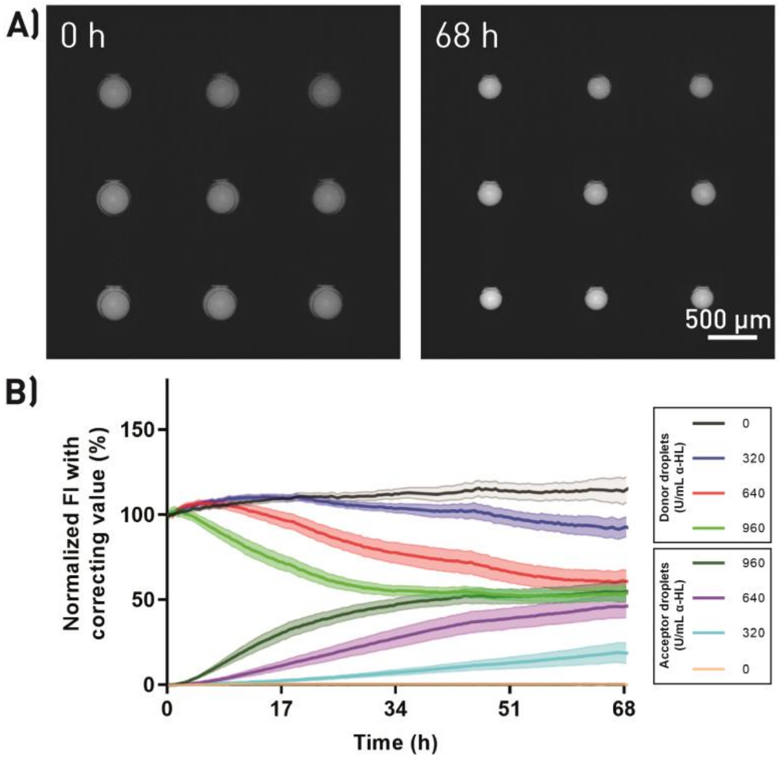


*Figure S3: A) Shrinkage of Alexa488 donor droplets connected to acceptor droplets without alpha-hemolysin (α-HL) over 68 h. The diameter decreased down to ~70 % of the original diameter within 68 hours. B) Corrected graph of the translocation of Alexa488 across α-HL pores, when the shrinkage is considered. The mean fluorescence intensity (FI) of every data point in Figure 3E was corrected with the following equation: $FI_{corrected} = FI \times (-0.0044 \times t + 1)$. Data were collected every ~30 min, N = 27 droplet networks for every curve, α-HL was only present in the acceptor droplets. The error bands represent the standard deviation.*

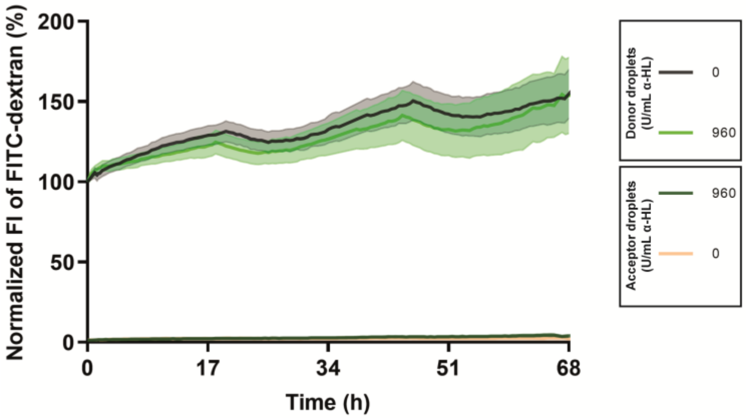


*Figure S4: Mean fluorescence intensity (FI) over time of 60 µM FITC-dextran translocation across α-HL pores. Data were collected every ~30 min, N = 27 droplet networks for every curve, α-HL was only present in the acceptor droplets. Both drops in FI correspond to the time points when 100 µL water was added to the plate holder to reduce droplet shrinkage. Most likely, a very small amount of FITC-dextran partitions into the oil and forms an equilibrium. When water is added without FITC-dextran, the equilibrium is adjusted (the effect of 100 µL is greater than that of the 25 nL droplets on the equilibrium). The error bands represent the standard deviation. The continuous increase in the normalized FI in the experiment can be attributed to slow droplet shrinkage over time. We only normalized to the starting fluorescence of the individual donor droplets and did not account for droplet shrinkage in the data normalization process.*

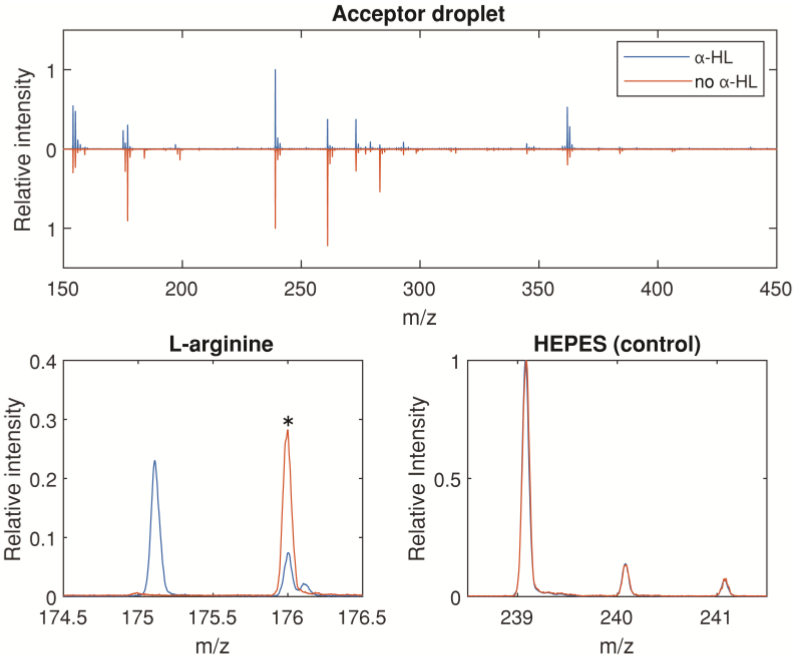


*Figure S5: MALDI-MS analysis of accepter droplets. The alpha-hemolysin (α-HL) dependence of membrane translocation is observed by MALDI-MS analysis for L-arginine (175.1 m/z). The acceptor droplet that contained α-HL (top graph) and did not (bottom flipped graph) is displayed for each condition. Data are shown for N = 1 droplet network. In addition, we pooled N = 7 droplet networks and received a similar result (data not shown). The isotopic pattern is for each analyte of interest and HEPES - as a positive control - is zoomed in on. Background peaks on the zoomed in images are denoted with a *. The matrix used was DHB. Each spectra is normalized to the intensity of the internal control HEPES (239.1 m/z).*

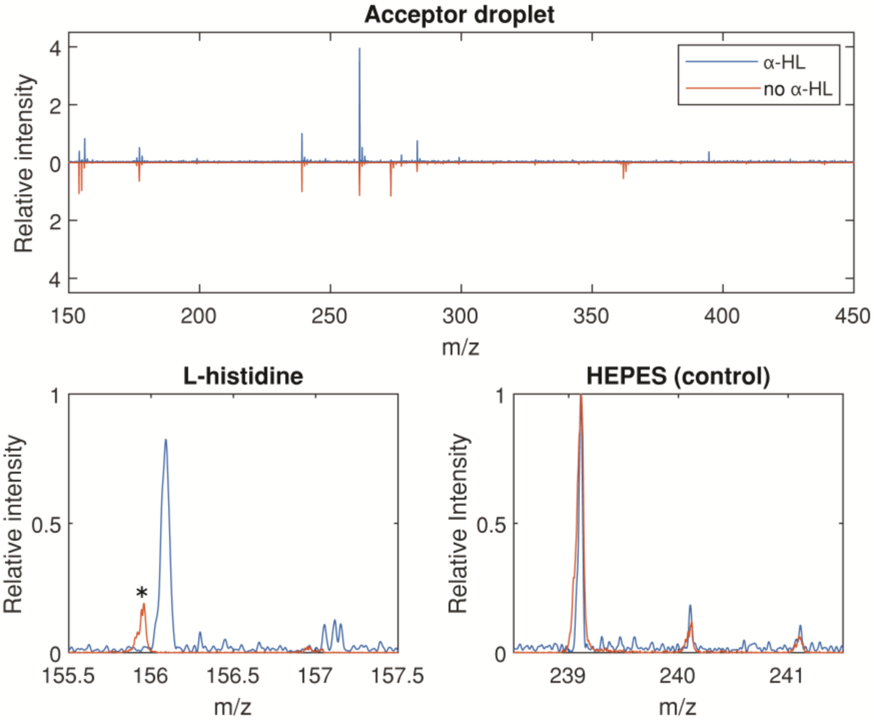


*Figure S6: MALDI-MS analysis of acceptor droplets. The alpha-hemolysin (α-HL) dependence of membrane translocation is observed by MALDI-MS analysis for L-histidine (156.1 m/z). The acceptor droplet that contained α-HL (top graph) and did not (bottom flipped graph) is displayed for each condition. Data are shown for N = 7 pooled droplet networks. The isotopic pattern is for each analyte of interest and HEPES - as a positive control - is zoomed in on. Background peaks on the zoomed in images are denoted with a *. The matrix used was DHB. Each spectra is normalized to the intensity of the internal control HEPES (239.1 m/z).*

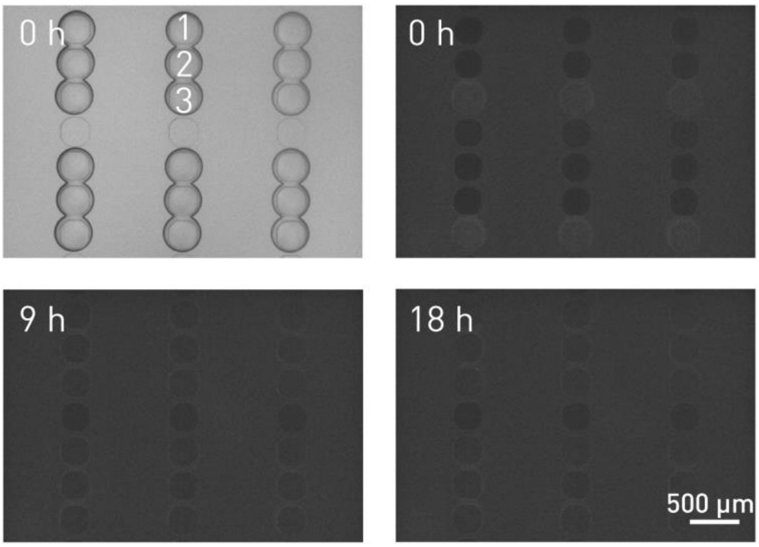


*Figure S7: Compartmentalized enzymatic cascade reaction negative control. No alpha-hemolysin was present. The lactose/lactase droplet (1) was added to the glucose oxidase droplet (2) ~180 min before the luminol droplet (3). The imaging starting point was directly after spotting of the luminol droplet. Three droplets in a network: Lactose and lactase in the top droplet (1). Glucose oxidase in the middle droplet (2). Luminol in the bottom droplet (3). We could not detect the formation of 3-aminophthalic acid (N = 42 droplet networks).*

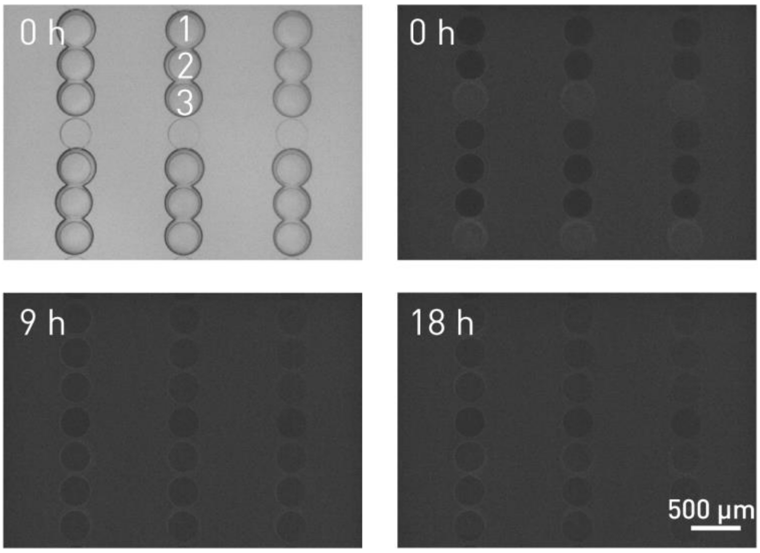


*Figure S8: Compartmentalized enzymatic cascade reaction negative control. No lactase was present. The lactose droplet (1) was added to the glucose oxidase droplet (2) ~120 min before the luminol droplet (3). The imaging starting point was directly after spotting of the luminol droplet. Three droplets in a network: Lactose with alpha-hemolysin (α-HL) in the top droplet (1). Glucose oxidase in the middle droplet (2). Luminol in the bottom droplet (3). We could not detect the formation of 3-aminophthalic acid (N = 42 droplet networks).*

## Rate constants of fluorophore translocation across alpha-hemolysin pores

In our fluorophore translocation studies (Figure 3), we were able to image the translocation process over a long time period. To extract more quantitative data, we calculated the rate constants ($k$). The rate constants describe the fraction of translocated fluorophores that are transferred over the alpha-hemolysis pore per time. For this, we first normalized the data to 100% of the starting fluorescence intensity of the individual donor plus acceptor droplets in the first image. In a second step, we additionally normalized every data point to 100% (fluorescence intensity of the donor plus acceptor droplets equals 100%). In third step, we removed the first 3 h to account for the lag in pore formation. Equation 1 and 2 allow for extracting kinetic rate constants by plotting the mass of translocated fluorophores in the acceptor ($M_a$) and donor droplet ($M_d$) over time ($t$) [1]. This exchange is reversible, so it is dependent on the rate constants $k_{da}$ (donor to acceptor) and $k_{ad}$ (acceptor to donor). The time ($t$) is corrected by $c$ to take into account both a delay in measurement start and a potential lag phase in the translocation process due to pore formation. Equations 1 and 2 were fitted to the data with OriginPro (2019, 9.6, OriginLab Corporation). The iteration algorithm was Levenberg Marquardt. Data from every droplet pair was individually fitted and evaluated. Table S1 shows the calculated rate constants and correction values.

$$M_a(t) = \left(-\frac{k_{ad}}{k_{da}+k_{ad}} \times \mathrm{e}^{-(k_{da}+k_{ad}) \times (t+c)} + \frac{k_{ad}}{k_{da}+k_{ad}}\right) \times 100\% \quad (1)$$

$$M_d(t) = \left(\frac{k_{da}}{k_{da}+k_{ad}} \times \mathrm{e}^{-(k_{da}+k_{ad}) \times (t+c)} + \frac{k_{ad}}{k_{da}+k_{ad}}\right) \times 100\% \quad (2)$$

*Table S1: Rate constants (k) and correction values (c) for the translocation of NBD-F, riboflavin, and Alexa488 across alpha-hemolysin (α-HL) pores. For every condition, the individual data of N = 27 connecting donor/acceptor droplet pairs was fitted with OriginPro. The rate constants $k_{da}$ (donor to acceptor) and $k_{ad}$ (acceptor to donor) describe the amount of fluorophore that is transferred over the α-HL pore. All values are noted as mean with the standard deviation (SD).*

| Fluorophore | alpha-hemolysin (U/ml) | | $k_{da} \times 10^{-3}$ (1/h) | | $k_{ad} \times 10^{-3}$ (1/h) | | c (h) | |
|---|---|---|---|---|---|---|---|---|
| | Donor | Acceptor | Mean | SD | Mean | SD | Mean | SD |
| NBD-F | 0 | 320 | 62.9 | 8.0 | 62.9 | 8.0 | -1.0 | 0.2 |
| NBD-F | 320 | 320 | 103.7 | 11.5 | 103.7 | 11.5 | -0.7 | 0.4 |
| NBD-F | 640 | 0 | 97.7 | 10.7 | 97.7 | 10.7 | 0.2 | 0.4 |
| NBD-F | 0 | 640 | 110.0 | 9.0 | 110.0 | 9.0 | -1.2 | 0.3 |
| NBD-F | 0 | 960 | 180.9 | 32.6 | 180.9 | 32.6 | -0.9 | 0.5 |
| Riboflavin | 0 | 320 | 3.7 | 1.2 | 3.7 | 1.2 | -3.2 | 1.8 |
| Riboflavin | 0 | 640 | 19.5 | 3.2 | 19.5 | 3.2 | -2.8 | 0.5 |
| Riboflavin | 0 | 960 | 137.0 | 30.7 | 137.0 | 30.7 | -0.2 | 0.5 |
| Alexa488 | 0 | 320 | 3.1 | 0.9 | 3.1 | 0.9 | -3.4 | 4.1 |
| Alexa488 | 0 | 640 | 14.8 | 4.6 | 14.8 | 4.6 | -5.6 | 0.8 |
| Alexa488 | 0 | 960 | 38.3 | 6.1 | 38.3 | 6.1 | -3.6 | 0.4 |

## Label-free molecule detection: Detected and theoretical monoisotopic peaks

*Table S2: Theoretical and observed monoisotopic H+ m/z.*

| Analyte | Detected m/z | Theoretical monoisotopic H+ m/z | Error in m/z |
|---|---|---|---|
| L-histidine | 156.0868 | 156.0768 | 0.0101 |
| L-arginine | 175.1151 | 175.1190 | -0.0039 |
| HEPES | 239.1104 | 239.1060 | 0.0044 |
| Riboflavin | 377.1206 | 377.1456 | -0.0249 |

## Supplementary Reference


1. Krämer SD. Quantitative aspects of drug permeation across in vitro and in vivo barriers. *European Journal of Pharmaceutical Sciences* **87**, 30-46 (2016).
