# Supplementary material for: Microfluidic platform enables tailored translocation and reaction cascades in nanoliter droplet networks
Source: Commun Biol. 2020 Dec 14;3:769. doi: 10.1038/s42003-020-01489-w (PMC7736871; doi:10.1038/s42003-020-01489-w)
Supplement: Supplementary file 2 — Description of Additional Supplementary Files [file 42003_2020_1489_MOESM2_ESM.pdf]

Description of Additional Supplementary Files

File Name: Supplementary Movie 1
Description: Spotting “ETH”. The video demonstrates the spotting process (10 fps, 4x).

File Name: Supplementary Movie 2
Description: Translocation Ca^{2+} across droplet network. The video shows a droplet network, droplet interface bilayers containalpha-hemolysin poresand the dropletsare filled with a calcium sensing dye (Fluo-4). The translocation of Ca^{2+} from the first droplet (without Fluo-4) is visible by the increase of fluorescence (10 fps, image every 5 minutes)

File Name: Supplementary Movie 3
Description: Demonstration of the three-step enzymatic reaction (7 fps, image every 15 minutes)
